# A Knock-In Mouse Model for the R120G Mutation of αB-Crystallin Recapitulates Human Hereditary Myopathy and Cataracts

**DOI:** 10.1371/journal.pone.0017671

**Published:** 2011-03-18

**Authors:** Usha P. Andley, Paul D. Hamilton, Nathan Ravi, Conrad C. Weihl

**Affiliations:** 1 Department of Ophthalmology and Visual Sciences, Washington University School of Medicine, St. Louis, Missouri, United States of America; 2 Department of Research, Veterans Affairs Medical Center, St. Louis, Missouri, United States of America; 3 Department of Neurology, Washington University School of Medicine, St. Louis, Missouri, United States of America; California State University Fullerton, United States of America

## Abstract

An autosomal dominant missense mutation in αB-crystallin (αB-R120G) causes cataracts and desmin-related myopathy, but the underlying mechanisms are unknown. Here, we report the development of an αB-R120G crystallin knock-in mouse model of these disorders. Knock-in αB-R120G mice were generated and analyzed with slit lamp imaging, gel permeation chromatography, immunofluorescence, immunoprecipitation, histology, and muscle strength assays. Wild-type, age-matched mice were used as controls for all studies. Both heterozygous and homozygous mutant mice developed myopathy. Moreover, homozygous mutant mice were significantly weaker than wild-type control littermates at 6 months of age. Cataract severity increased with age and mutant gene dosage. The total mass, precipitation, and interaction with the intermediate filament protein vimentin, as well as light scattering of αB-crystallin, also increased in mutant lenses. In skeletal muscle, αB-R120G co-aggregated with desmin, became detergent insoluble, and was ubiquitinated in heterozygous and homozygous mutant mice. These data suggest that the cataract and myopathy pathologies in αB-R120G knock-in mice share common mechanisms, including increased insolubility of αB-crystallin and co-aggregation of αB-crystallin with intermediate filament proteins. These knock-in αB-R120G mice are a valuable model of the developmental and molecular biological mechanisms that underlie the pathophysiology of human hereditary cataracts and myopathy.

## Introduction

αB-Crystallin is a member of the small heat-shock protein family, which consists of 10 proteins in humans [1]. The αB-crystallin protein has a subunit mass of 20 kDa but forms molecular aggregates with a mass of approximately 650 kDa [2]. It is abundantly expressed in the eye lens fiber cells, where it is associated with the closely related protein αA-crystallin [3], and is also constitutively expressed at significant levels in heart and skeletal muscle and lens epithelial cells [4]–[6]. αB-crystallin is a functional chaperone protein that can bind to denatured substrate proteins, thereby preventing their non-specific aggregation [5]. It is upregulated in several pathologic conditions where, as a molecular chaperone, it is thought to provide a first line of defense against misfolded or aggregation-prone proteins [7]. αB-crystallin has received significant attention in recent years because it has been linked to muscle and neurological disorders, as well as immunity and cancer [8]–[13]. However, how αB-crystallin contributes to these pathologies is not clearly understood.

Hereditary cataracts exhibit diverse etiology and morphology [14]. Cataracts may be inherited by an autosomal recessive, autosomal dominant, or X-linked mechanism [15]. Cataracts caused by missense mutations in crystallin genes are most commonly autosomal dominant disorders [16]. Understanding the pathophysiology of hereditary cataracts can yield insight into the mechanisms of cataractogenesis in general [17]. However, the relationships between cataract etiology, lens morphology, and the underlying molecular mechanisms that control lens structure and function are currently unclear [16]–[18].

Numerous crystallin gene mutations have been reported to be associated with hereditary cataracts [12], [19]–[21]. Mutations in the αB-crystallin gene cause either isolated cataracts or cataracts associated with myopathy. For example, the αB-crystallin mutation R120G is associated with cataracts and desmin-related myopathy (DRM), a disorder of the skeletal muscle [12]. In contrast, αB-crystallin Q151X and 464delCT mutations are linked to DRM, but not to cataracts [22]. In addition, the αB-crystallin R157H mutation has been linked to cardiomyopathy [23], while the P20S, D140N, and 450delA mutations are associated with hereditary human cataracts [24]–[26]. While the characterization of the effects of most αB-crystallin mutations is limited, the effects of the R120G mutation on protein structure and chaperone activity have been extensively investigated [27]–[30]. Both *in vitro* studies of recombinant mutant αB-crystallin and transgenic models expressing the mutant protein *in vivo* have contributed to our understanding of the effect of this mutation on protein function [27], [31]–[33]. Using *in vitro* recombinant substrate proteins, chaperone assays have indicated that αB-R120G reduces or abolishes chaperone function, becomes unstable and prone to aggregation and insolubilization with time, and exists as a large oligomer with a molecular mass twice that of wild-type αB-crystallin [31], [34]. The loss of chaperone function leads to aggregation of intermediate filament proteins with the mutant αB-crystallin and the formation of inclusion bodies in cells [31].

Patients harboring the αB-R120G mutation experience symptoms of muscle weakness, cardiomyopathy, and cataracts. The autosomal dominant A→G mutation in codon 120 of exon 3 of *CRYAB* leads to substitution of arginine to glycine [12]. The arginine residue at position 120 in the αB-crystallin amino acid sequence is highly conserved and has been shown to be essential for the quaternary structure and functional integrity of human αB-crystallin [27]. This residue lies in the C-terminal region that is crucial for the solubilization and chaperone functions of αB-crystallin [27]. Mutation of this residue causes a loss of the chaperone activity of αB-crystallin *in vitro*, promotes interaction between the mutant protein and the type III intermediate filament protein desmin [28], and is associated with adult onset myopathy and accumulation of desmin in humans [12]. Interestingly, αB-crystallin/HSPB2 gene knockout mice exhibit myopathy but do not develop cataracts [8].

Several mechanisms have been proposed to explain DRM disease etiology, including altered protein processing [35], [36] and the loss of αB-crystallin chaperone function [27]. An alternative mechanism involves alteration of the binding between αB-crystallin and desmin filaments. The R120G mutation enhances the binding capacity of αB-crystallin for desmin and decreases their dissociation constant. The desmin aggregates that are characteristic of the disease histopathology might result from direct alterations of the interaction of αB-R120G with desmin filaments [28]. The loss of αB-crystallin function may also decrease cell viability because αB-crystallin negatively regulates apoptosis by inhibiting caspase 3 activation [37]. Additionally, αB-crystallin has been shown to inhibit cytochrome c- and caspase 8-dependent autoproteolytic maturation and activation of caspase 3 [38]. Furthermore, αB-crystallin inhibits Ras-induced apoptosis [39]. While αB-crystallin is known to function in these protein processing, chaperone, and apoptosis pathways *in vitro*, whether these pathways are involved in the development or progression of myopathy and cataracts remains unclear.

Currently, no *in vivo* model exists to explore the effect of the R120G mutation in lenses or skeletal muscle. Furthermore, no photographic or digital image documentation of human cataracts caused by the R120G mutation is available. To explore how lens opacities develop over time and to examine the spatial localization of the opacities, we generated heterozygous knock-in mice carrying the R120G mutation. The advantage of the knock-in approach is that the effects of the mutation can be studied in every cell under the control of the endogenous promoter, making it possible to analyze the phenotype in the mouse lens and determine whether it mimics human disease [40]–[42]. Additionally, the creation of homozygous mutant mice by interbreeding allows the phenotypic comparison of heterozygous and homozygous mice and the study of gene-dosage effects of the mutation. The results of our study show that the R120G mutation causes cataracts at a young age, and some mice also develop corneal opacity and small eye/lens phenotypes. The knock-in mutant lenses exhibited aberrant morphology, crystallin aggregation, and altered protein-protein interactions between αB-crystallin and the intermediate filament protein vimentin. We also demonstrated that the R120G knock-in mice develop myopathy, weakness, loss of αB-crystallin solubility, and an increase in desmin-αB-crystallin aggregates in muscle tissue. Thus, the αB-crystallin R120G knock-in mouse model recapitulates the key symptoms of human hereditary myopathy and cataract disease pathology.

## Results

### Generation of αB-R120G mutant knock-in mice

**Figure 1:**
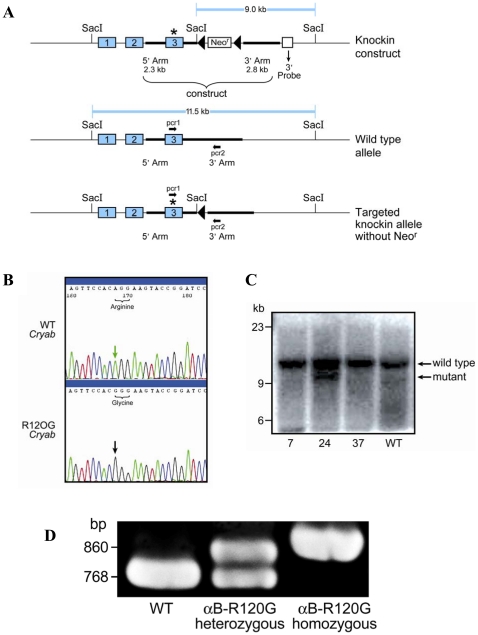
Gene-targeting strategy and genotype analysis. *A)* Diagram illustrating the gene-targeting strategy used to produce αB-R120G gene knock-in mice. The numbered rectangles represent exons and the starred exon 3 indicates the mutated exon. Restriction sites relevant to Southern blot analysis are shown together with the size of the restriction fragments. Lox P sites are represented as closed triangles. The 3′ probe that was used for Southern blot analysis is shown below the mutated allele. Neo^r^ represents the neomycin cassette, which was excised by homologous recombination after introduction of the αB-R120G knock-in plasmid and the Turbo-Cre plasmid. Bold arrows indicate the PCR primers (pcr1 and pcr2) used to detect the wild-type and mutated alleles. *B)* Genomic DNA from ES clones was sequenced to verify the A to G mutation in the mouse αB-crystallin gene. *C)* Southern blot of *Sac*I digests showing clones 7, 24, 37, and a wild-type clone with no insertion. Of these, clone 24 demonstrated insertion of the plasmid containing neomycin *(mutant)*. The lower band (9.0 kb) represents the correctly targeted ES clone containing the insertion. The native αB-crystallin gene (11.5 kb) was present in each positive clone. *D)* PCR screening of mouse genomic tail DNA confirmed recombination of the Lox P sites showing that neomycin was deleted. At the 5′ end, a sense flanking primer (pcr1) was paired with an antisense αB-crystallin gene intronic primer (pcr2). These primers amplified a 768-bp band from the wild-type (WT) αB-crystallin gene compared with an 860-bp band from neomycin-deleted knock-in chromosomes. Both the 768- and 860-bp bands were amplified in heterozygous mice. Absence of the 768-bp band and detection of only the 860-bp band indicated homozygosity for the αB-R120G mutation.

**Figure 2:**
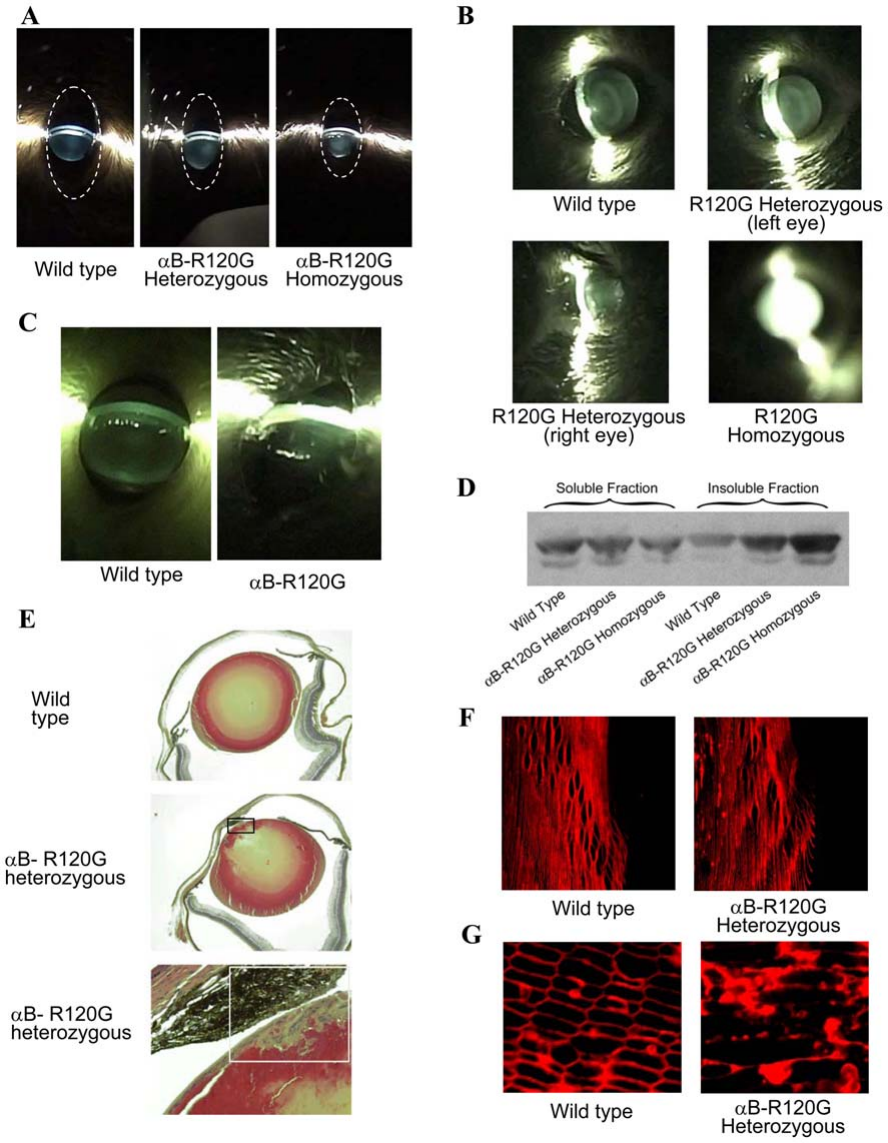
Eye and lens phenotypes of αB-R120G knock-in mice. Eyes were dilated in non-anesthetized mice and examined using a slit lamp. *A)* Slit lamp micrographs of 5-week-old mice. *Left panel*, wild-type mice with clear lenses. *Middle panel*, heterozygous αB-R120G knock-in mice displaying low-grade opacities in the nuclear and posterior regions of the lens. *Right panel*, homozygous αB-R120G knock-in mice with prominent opacity in the lens nucleus (stage 3) and the small eye phenotype. The dotted line shows the outline of the eye. *B)* Lens opacity and corneal abnormality in 10-month-old αB-R120G mouse eyes. *Top left*, wild-type. *Top right*, left eye. *Bottom left*, right eye of an αB-R120G heterozygous mutant mouse. *Bottom right*, eye of homozygous mutant mouse showing complete (stage 4) opacity. *C)* Corneal abnormality in αB-R120G heterozygous mutant mouse eye. The wild type eye was normal. *D)* Immunoblot analysis of lens water-soluble and water-insoluble proteins using an antibody specific for αB-crystallin. *E)* Histological analysis of αB-R120G heterozygous lenses. Lens sections were stained with hematoxylin and eosin (H&E). *Top panel*, wild-type lens with normal epithelial and fiber cell morphology. *Middle panel*, an αB-R120G heterozygous lens with a multilayered region of cells. *Lower panel*, a higher magnification image of the multilayerd region shown in the rectangle of the *middle panel*. *F, G)* Immunofluorescence analysis of major intrinsic protein (MIP) expression in wild-type and αB-R120G heterozygous knock-in lenses. *F)* Mid-sagittal lens sections stained with anti-MIP (AQP0) to visualize fiber cell membranes. Equatorial region of the lens. Sections of wild-type *(left panel)* and homozygous *(right panel)* lenses are shown. Visualization of fiber cell membranes at the onset of differentiation (cell elongation) region. *G)*. Cross-sections of anterior cortical fibers of the lens. Sections of wild-type *(left panel)* and heterozygous *(right panel)* lenses are shown.

To investigate the effect of the αB-R120G mutation in mice, we generated αB-R120G knock-in alleles (**Fig. 1** ***A*** **–** ***D***). Germline heterozygous alleles were generated by crossing founder mice carrying the R120G mutation on one allele with C57BL6 mice. Polymerase chain reaction (PCR) genotyping of litters generated from crosses of heterozygotes resulted in the expected amplification products (Fig. 1*D*). The αB-R120G knock-in mice bred normally and produced offspring in the anticipated Mendelian ratio, indicating that the mutation was not embryonic lethal. αB-R120G heterozygous and homozygous mice were viable and exhibited no obvious phenotype at birth. Lens opacities according to age and genotype are shown in **Fig. 2** ***A–C*** and **Table 1**. αB-R120G mice displayed a complex ocular phenotype when examined by slit lamp biomicroscopy. Of the heterozygous mutant mice (n = 37), 84% had lens opacities, with the stage of cataract increasing with age (Table 1). Cataract stage increased from 1.4±0.6 at 3–8 weeks to 2.6±0.8 at 40–56 weeks (p<0.0001) (**Tables 1** **and** **2**). Wild-type mice displayed a low-grade haze or opacity (stage 0–0.5) up to 20 weeks, and stage 1 opacities at >40 weeks manifested as a ring in the center of the lens. In contrast, homozygous mutant mice had cataract stages of 1.2±0.9 (3–8 weeks), 2.6±0.9 (12–20 weeks), and 3.2±1.2 (40–56 weeks; p = 0.005, 3–8 weeks versus 40–56 weeks). The cataract stage increased with gene dosage at 12–20 weeks for heterozygous and homozygous mutants (1.7±1.0 and 2.6±0.5, respectively; p = 0.02). However, the percent of mice with cataracts did not increase with gene dosage. Wild-type mice (n = 21) did not have small eyes or corneal abnormalities. In contrast, at all ages combined, 22% and 21% of heterozygous and homozygous mice, respectively, had small eyes (Fig. 2*A–C*). The small eye phenotype was not gene dosage-related. Additionally, corneal abnormalities and small eyes were not bilateral (Fig. 2*B*). Wild-type mice (n = 21) did not show corneal abnormalities or small eyes. However, 2/37 (5%) heterozygous mutant mice and 3/19 (16%) homozygous mutant mice developed corneal abnormalities.

**Table 1:** Eye abnormalities in αB-R120G knock-in mice.

Age (weeks)	3–8		12–20		40–56		Total (all ages combined)
**Genotype**	**Cataract (lens opacities)**						
		Cataract stage		Cataract stage		Cataract stage	
WT	2/10 (20%)	0–0.5(0.3±0.3)	1/4 (25%)	0–0.5(0.6±0.6)	4/7 (57%)	1–1.5(1.2±0.3)	7/21 (33%)
Het	17/17 (100%)	1–3(1.4±0.6)	6/10 (60%)	0.5–3(1.7±1.0)	10/10 (100%)	2–4(2.6±0.8)	31/37 (84%)
Homo	8/10 (80%)	0–2(1.2±0.9)	5/5 (100%)	2–4(2.6±0.9)	3/4 (75%)	3–4(3.2±1.2)	16/19 (84%)
	**Small eye and lens**						
WT	0/10 (0%)		0/4 (0%)		0/7 (0%)		0/21 (0%)
Het	5/17 (29%)		0/10 (0%)		3/10 (30%)		8/37 (22%)
Homo	2/10 (20%)		0/5 (0%)		2/4 (50%)		4/19 (21%)
	**Corneal abnormalities**						
WT	0/10 (0%)		0/4 (0%)		0/7 (0%)		0/21 (0%)
Het	2/17 (12%)		0/10 (0%)		0/10 (0%)		2/37 (5%)
Homo	1/10 (10%)		0/5 (0%)		2/4 (50%)		3/19 (16%)

WT, wild-type; Het, heterozygous; Homo, homozygous.

**Table 2:** Statistical analysis of data in Table 1.

Age (weeks)	Genotype comparison	p	Age comparison	p
3–8	WT vs. Het	5.8×10^−11^*	WT 3 vs. 40 weeks	5.07×10^−7^*
	WT vs. Homo	0.013*	WT 12 vs. 40 weeks	0.005*
	Het vs. Homo	0.23	WT 3 vs. 12 weeks	0.09
12–20	WT vs. Het	0.0005*	Het 3 vs. 40 weeks	2.09×10^−6^*
	WT vs. Homo	0.0001*	Het 12 vs. 40 weeks	0.003*
	Het vs. Homo	0.022*	Het 3 vs. 12 weeks	0.13
40–56	WT vs. Het	2.36×10^−7^*	Homo 3 vs. 40 weeks	0.005*
	WT vs. Homo	0.007*	Homo 12 vs. 40 weeks	0.20
	Het vs. Homo	0.16	Homo 3 vs. 12 weeks	0.003*

Asterisks denote statistical significance with p<0.05.

WT, wild-type; Het, heterozygous; Homo, homozygous.

### Qualitative assessment of solubility of αB-crystallin in mutant knock-in lenses

Western blot analysis was performed to qualitatively assess the solubility of αB-crystallin in mutant lenses. The proportion of water-soluble αB-crystallin was lower in mutant lenses, whereas the proportion of insoluble αB-crystallin was higher in heterozygous mutant lenses than in wild-type lenses, and higher in homozygous mutant lenses than in heterozygous mutant lenses (Fig. 2*D*). These results demonstrate a gene-dosage effect of the αB-R120G mutation on lens αB-crystallin solubility.

### Morphological changes in αB-R120G knock-in lenses

In small eyes with corneal damage (the most severe phenotype), distinctive multilayering of the anterior lens epithelial cells into plaques was observed during histological analysis (Fig. 2*E*). Immunofluorescence analysis of lens sections revealed that the lens epithelial cells appeared to enter the fiber cell zone normally and fiber cell elongation appeared to occur (Fig. 2*F*). Thus, organization of fiber cells in the equatorial sections of αB-R120G heterozygous lenses likely occurred, but was apparently less uniform than the neat packing of membranes in wild-type lenses. In contrast, fiber cell membranes of anterior lens cortical fibers were not well organized in the αB-R120G heterozygous mutant lenses and exhibited highly disturbed membrane packing compared with wild-type lenses (Fig. 2*G*).

### Changes in molecular masses of proteins isolated from αB-R120G knock-in lenses

**Figure 3:**
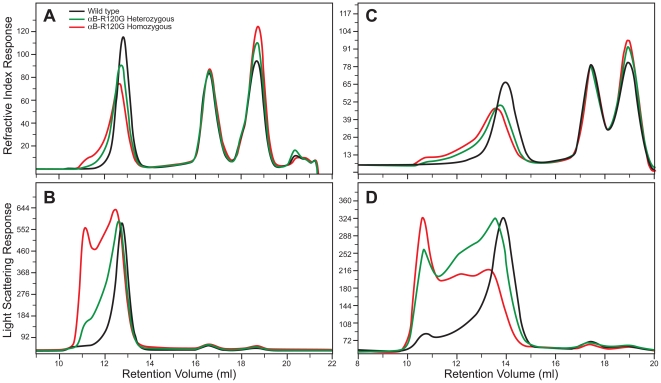
Size-exclusion gel permeation chromatography (GPC) analysis of lens proteins from αB-R120G knock-in mice. Lens proteins were separated into water-soluble and insoluble fractions, and then the water-soluble fractions were analyzed by GPC. *A, B)* Three-month-old lenses. *C, D)* Eight-month-old lenses. *A)* Refractive index profile of the water-soluble proteins in wild-type *(black)*, heterozygous mutant *(green)*, and homozygous mutant *(red)* lenses. *B)* Right-angle light scattering in proteins eluting from the size exclusion chromatography columns for wild-type *(black)*, heterozygous mutant *(green)*, and homozygous mutant *(red)* lens proteins. *C)* Refractive index profile of the water-soluble proteins from wild-type *(black)*, heterozygous mutant *(green)*, and homozygous mutant *(red)* lenses. *D)* Right-angle light scattering in proteins eluting from the size exclusion chromatography columns from wild-type *(black)*, heterozygous mutant *(green)*, and homozygous mutant *(red)* lens proteins. Note that while the y-axis of the light scattering intensity is lower in 8-month-old lens proteins *(D)*, it is spread out over 5 ml, compared with 3 ml in the 3-month-old lens proteins *(B)*. Thus, the area under the light scattering curve is greater at 8 months. Note also that the higher molecular weight for α-crystallin (Table 3) calculated by the software shows that the light scattering intensity with respect to the refractive index signal is higher in the 8-month-old than in the 3-month-old lens proteins.

Next we investigated the effects of the R120G αB-crystallin mutation on the sizes of αB-crystallin proteins using gel permeation chromatography (GPC) with light scattering and refractive index (RI) measurements. **Fig. 3** demonstrates the chromatography profiles of water-soluble lens protein from wild-type mice, and heterozygous and homozygous mutant mice. Fig. 3*A* shows the RI profile of lens crystallins from 3-month-old mice isolated by GPC. Based on previous studies [43], [44] and using immunoblot analysis of proteins eluted in column fractions (data not shown), the first peak was identified to be α-crystallin, the second to be β-crystallin, and the third to be γ-crystallin. The RI profile revealed an incremental loss of the α-crystallin fraction from the soluble phase (Fig. 3*A*). This decrease was accompanied by an incremental increase in the molecular mass of the α-crystallin fraction in the soluble phase from wild-type to heterozygous mutant to homozygous mutant lenses. An obvious increase also existed in γ-crystallin in the soluble fraction of mutant lenses with increasing mutant gene dosage (Fig. 3*A*). Fig. 3*B* shows a plot of light scattering measurements for proteins eluted from the column. As demonstrated previously [43], α-crystallin is the major contributor to light scattering. **Table 3** shows the effects of the αB-R120G mutation on the average molecular mass, viscosity, and hydrodynamic radius of the major crystallin peaks eluted from the GPC column. The average molecular mass of the α-crystallin fraction from 3-month-old mice was 1113 kDa for wild-type lenses, 1554 kDa for heterozygous mutant lenses, and 2632 kDa for homozygous mutant lenses. The hydrodynamic radius was 10.1 nm for wild-type α-crystallin, and 11.5 and 13.3 nm for heterozygous and homozygous mutant lenses, respectively. The molecular mass and hydrodynamic radius of α-crystallin increased significantly with age in mutant knock-in lenses, but not in wild-type lenses (Table 3). Fig. 3*C and D* show the refractive indices and 90° light-scattering profiles for mouse lens crystallins isolated from the water-soluble fractions of 8-month-old wild-type, αB-R120G heterozygous, and homozygous mutant knock-in mouse lens lysates. These data also demonstrate that the light scattering of mutant lens α-crystallin fractions increased dramatically with age. Additionally, higher molecular weights of β- and γ-crystallins were observed in 4-month-, 8-month-, and 9-month-old lenses (Table 3). At 9 months, there was a marked increase in aggregation of the β- and γ-crystallin fractions. The elevation in the γ-crystallin fraction was consistent for all 3-month-old mice (Fig. 3*A*) and 8-month-old mice (Fig. 3*C*), supporting the conclusion that the R120G mutation in αB-crystallin leads to increased abundance of γ-crystallin. We found that cleaved peptides of α- and β- crystallins (major lens soluble proteins) were not contaminating our assessment of γ-crystallin in mutant lenses because immunoblot analysis using antibodies against the total α- and β-crystallin fractions revealed that α-crystallin and β-crystallin are at trace levels in the γ-crystallin fractions (data not shown).

**Table 3:** Molecular weight (M_w_) and hydrodynamic radius (R_h_) of crystallins in αB-R120G mice.

Age (months)	Genotype	M_w_ (kDa)	R_h_ (nm)	M_w_ (kDa)	R_h_ (nm)	M_w_ (kDa)	R_h_ (nm)
		α-Crystallin		β-Crystallin		γ-Crystallin	
3	WT	1113×10^3^	10.122	50.717	3.79	21.683	2.63
4	WT	1130×10^3^	10.473	53.015	4.020	20.175	2.495
8	WT	1412×10^3^	11.059	61.888	3.842	21.823	2.587
9	WT	2122×10^3^	11.389	70.677	3.371	26.737	2.731
3	αB-R120G Het	1154×10^3^	11.47	50.820	3.537	22.720	2.419
4	αB-R120G Het _(group 1)_	2014×10^3^	13.462	61.589	4.054	23.179	2.491
4	αB-R120G Het _(group 2)_	1592×10^3^	12.085	56.472	3.573	20.801	2.474
9	αB-R120G Het	6305×10^3^	16.637	168.929	4.769	47.070	3.155
3	αB-R120G Homo	2632×10^3^	13.292	67.279	4.022	25.216	2.376
4	αB-R120G Homo _(group 1)_	2855×10^3^	13.974	75.913	3.592	20.223	2.331
4	αB-R120G Homo _(group 2)_	2749×10^3^	14.600	70.269	4.899	23.853	2.540
8	αB-R120G Homo	3249×10^3^	14.387	86.584	3.931	32.316	2.828
9	αB-R120G Homo	*	**	167.085	**	46.205	**

*Proteins from completely opaque 9-month-old homozygous mutant lenses were largely water-insoluble. The estimated molecular weight of the α-crystallin fraction was >10×10^6^. Most of the lens proteins did not go through the column filter (0.2-µm). The concentration of protein that went through the column was very low (0.128 mg/ml). Insufficient soluble protein made it difficult to accurately evaluate the R_h_ value from viscosity measurements.

**Protein concentration of the homozygous lens proteins was too low for an accurate determination of R_h_. Data for two independent sets of 4-month-old mutant lenses (group 1 and group 2) demonstrate minor differences between litters.

WT, wild-type; Het, heterozygous; Homo, homozygous.

### Alteration of lens intermediate filament protein vimentin in αB-R120G knock-in mice

**Figure 4:**
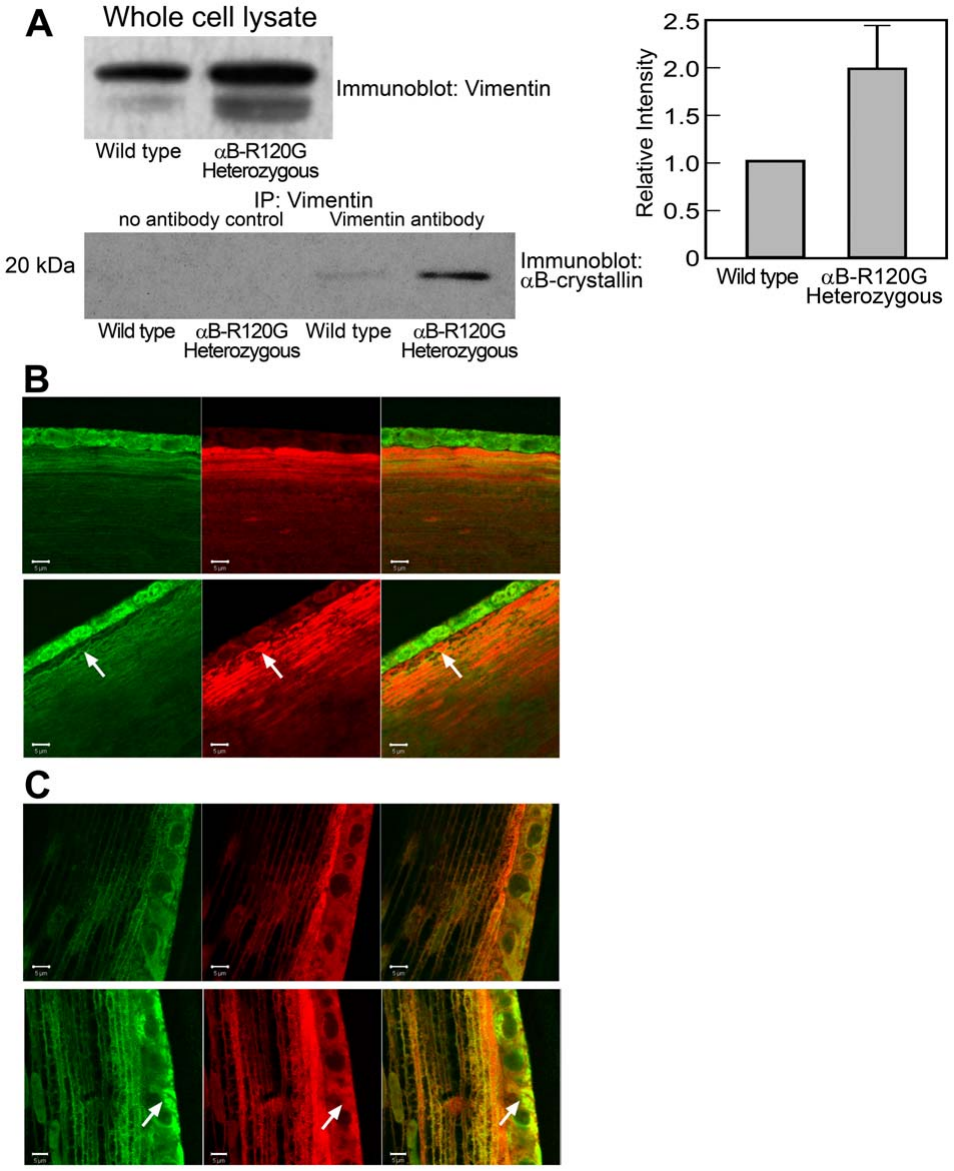
Interaction of αB-crystallin with vimentin in wild-type and αB-R120G knock-in lenses. *A)* Twelve-week-old lens protein extracts were immunoprecipitated with anti-vimentin. Two to four lenses were used per experiment. Immunoprecipitates were immunoblotted using antibodies specific for αB-crystallin. Controls for immunoprecipitation included rabbit serum containing no primary antibody (no antibody controls). Whole cell lysates were also analyzed to assess the total amount of vimentin and αB-crystallin in the lysates. αB-crystallin levels were identical in wild-type and heterozygous whole cell lysates (data not shown). Densitometric scans were obtained, and immunoprecipitated αB-crystallin and whole cell lysate vimentin was determined. Bar graphs to the right show the average of two independent experiments. *B, C)* Immunohistochemical assessment of co-localization of vimentin and αB-crystallin in αB-R120G mutant lenses. Mid-sagittal lens sections (4 µm) were stained with antibodies against vimentin (green) and αB-crystallin (red) and visualized using a confocal microscope. *B)* Anterior cortical fibers. *Top row*, wild-type lens. *Bottom row*, αB-R120G heterozygous lens. Arrows indicate the areas of dysmorphology. *C)* Equatorial lens epithelium and fibers. *Top row*, wild-type lens. *Bottom row*, αB-R120G heterozygous lens. Note that the heterozygous mutant lenses showed more clusters of vimentin and αB-crystallin *(arrows)*. Four wild type and four αB-R120G heterozygous mutant lenses were analyzed.

Previous studies have suggested that the αB-R120G mutation causes DRM and cataracts due to faulty interactions between αB-crystallin and intermediate filament proteins such as vimentin [12], [31]. To investigate the mechanisms of lens opacity formation in the knock-in lenses, we assessed the interactions between mutant αB-crystallin and vimentin. Immunoprecipitation using an antibody specific for vimentin followed by immunoblotting with αB-crystallin-specific antibodies revealed that more αB-crystallin interacted with vimentin in heterozygous mutant lenses than in wild-type lenses (**Fig. 4** ***A***). Densitometric analysis showed that the amount of vimentin also increased in the whole cell lysate of heterozygous mutant lenses compared with the wild type. This suggests that the mutant lenses have a higher concentration of vimentin.

We also assessed the interaction of vimentin with mutant αB-crystallin by immunofluorescence of lens epithelial and cortical fiber cells, which express high levels of vimentin (Fig. 4*B*). We observed increased clusters of mutant αB-crystallin and vimentin, indicating aggregation. We also observed co-aggregates of αB-crystallin and vimentin in many of the heterozygous mutant lens epithelial and cortical fiber cells (Fig. 4*C*).

### αB-R120G knock-in mice develop myopathy with αB-crystallin inclusions

**Figure 5:**
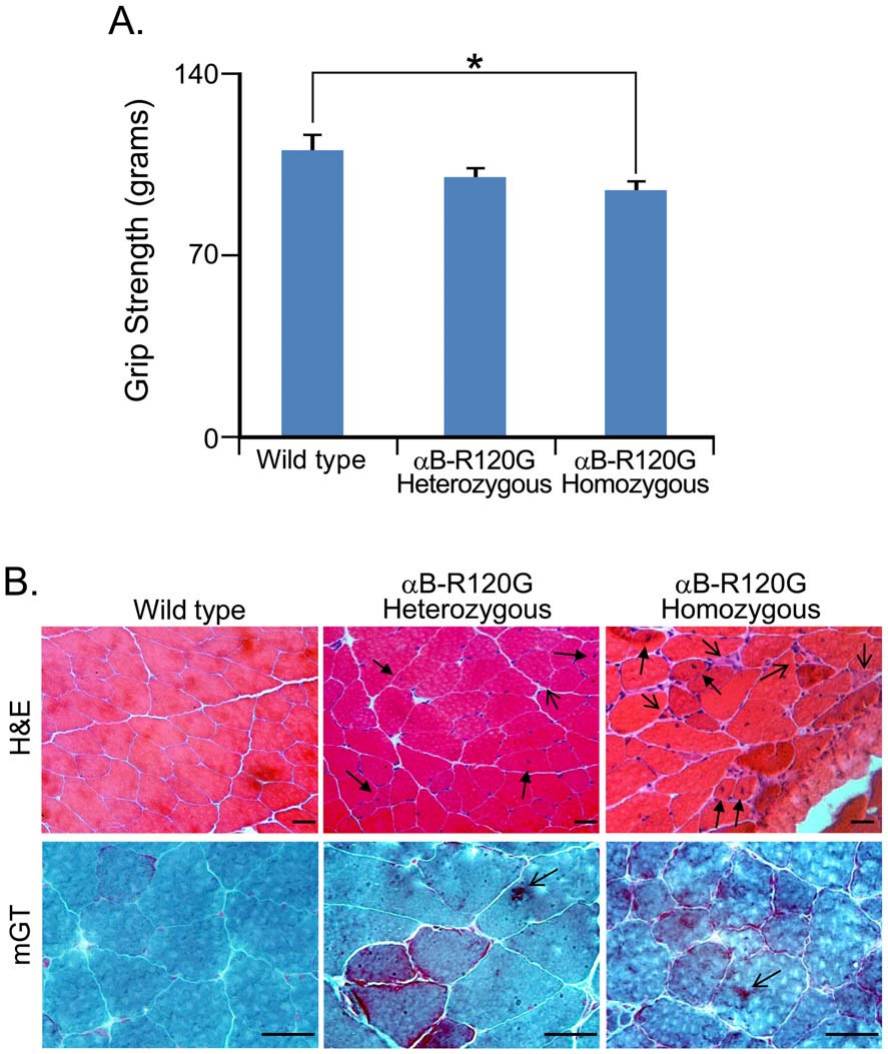
*In vivo* strength and muscle histopathology in αB-R120G knock-in mice. *A*) Average grip strength of αB-R120G heterozygous (n = 27), homozygous (n = 20), or wild-type (n = 10) control littermates at ∼6 months of age. *p<0.05. *B)* H&E or modified Gomori trichrome (mGT) staining of tibialis anterior (TA) muscle from 10-month-old wild-type, αB-R120G heterozygous, or αB-R120G homozygous mutant mice. *H&E*, evidence of myopathy in mutant muscle with small angular fibers (*open arrowhead*) and internal nuclei (*closed arrowhead*). *mGT*, increased dark staining consistent with the accumulation of myofibrillar proteins (*arrows*) in mutant muscle. Scale bar is 50 µm.

**Figure 6:**
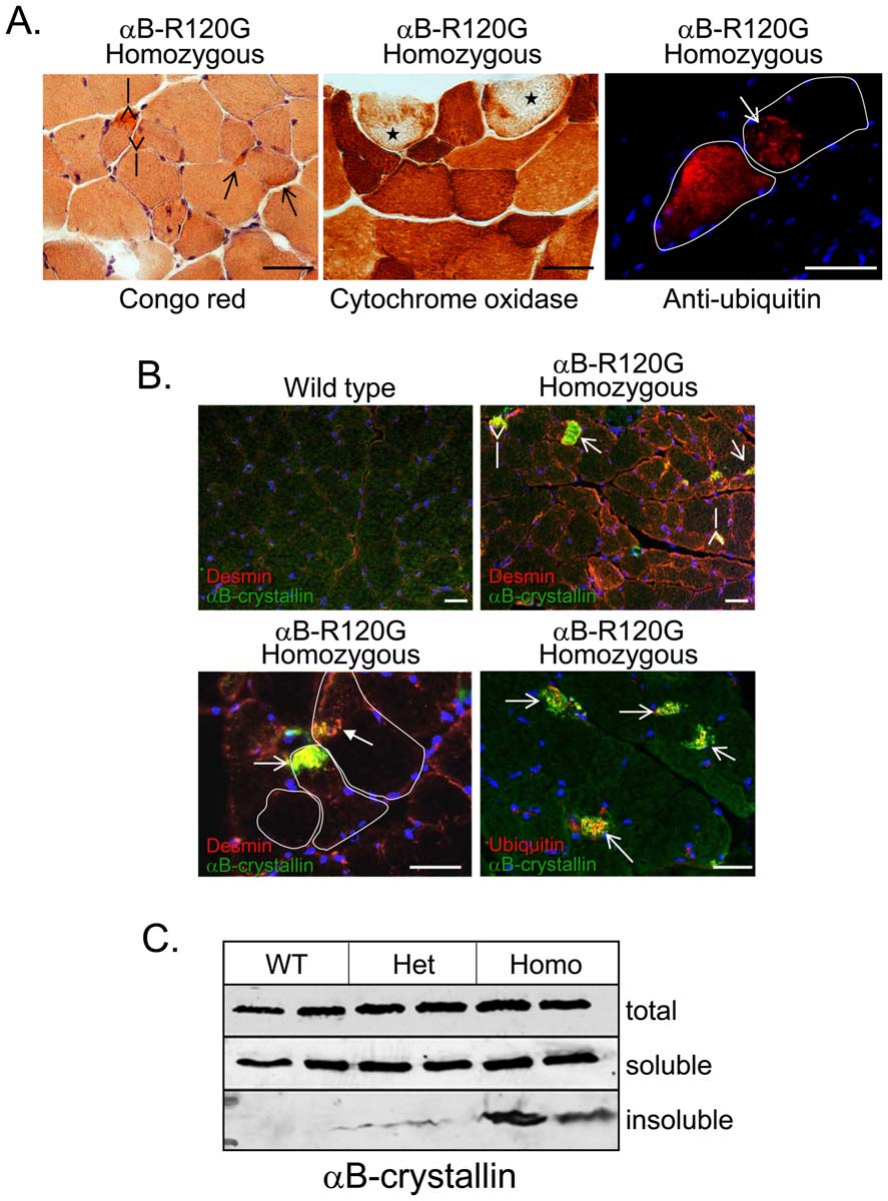
Histopathology and αB-crystallin insolubility in αB-R120G knock-in mice. *A)* Additional staining of 10-month-old αB-crystallin R120G homozygous mouse TA with Congo red shows multiple congophilic inclusions (*red; open arrows*). Cytochrome oxidase staining (*brown*) is absent or cleared from the central region of scattered myofibers (*starred fibers*). Immunofluorescence using an antibody against ubiquitinated proteins (*red*) highlights large ubiquitinated aggregates within scattered fibers. Blue staining is nuclear DNA. Scale bar is 50 µm. *B)* Triple immunofluorescence staining of TA muscle from 10-month-old wild-type or αB-R120G homozygous mutant mice using anti-αB-crystallin (*green*), anti-desmin (*red*), or anti-ubiquitin (*red in bottom right panel only*), and nuclear DNA (*blue*). Open arrowhead shows αB-crystallin and desmin co-aggregate, while closed arrowhead denotes isolated desmin inclusion. Individual myofibers are outlined in white. Scale bar is 50 µm. *C)* Immunoblot using αB-crystallin antibody of total, detergent-soluble, or detergent-insoluble proteins from TA muscle lysates of 4.5-month-old wild-type (*WT*), αB-R120G heterozygous (*het*), or homozygous (*homo*) mutant knock-in mice. Histopathology was performed on at least two mice per age.

Forelimb grip strength assessment was performed for αB-R120G heterozygous, homozygous mutant, and wild-type control littermates at 6 months of age. Strength was significantly lower in homozygous mice compared with wild-type controls (**Fig. 5** ***A***). Ten-month-old wild-type control littermates had normal muscle tissue structure when visualized with hematoxylin and eosin (H&E) or modified Gomori trichrome (mGT) staining (Fig. 5*B*). In contrast, H&E staining of the tibialis anterior muscle of heterozygous and homozygous mutant mice exhibited myopathy with dark basophilic fibers, internal nuclei, scattered necrosis, and fibrosis by 10 months of age, although the degree of myopathy was greater in homozygous mutant mouse muscle (Fig. 5*B*). αB-R120G heterozygous and homozygous mutant mice also displayed darker stained regions in mGT-stained muscle sections, consistent with myofibrillar disarray, than wild-type littermates (Fig. 5*B*). Additionally, αB-R120G homozygous mouse muscle had congophilic inclusions—“rubbed out” fibers—as visualized by cytochrome oxidase staining and ubiquitinated aggregates (**Fig. 6** ***A***). To evaluate constituents of the inclusions in mutant mouse muscle, we immunostained αB-R120G muscle tissue sections from homozygous mutant and wild-type control mice with antibodies against αB-crystallin and desmin. These proteins accumulated as inclusions in αB-R120G heterozygous and homozygous mutant muscle but not in wild-type muscle (Fig. 6*B*). αB-crystallin aggregates were ubiquitin-positive and often co-localized with large desmin inclusions in mutant muscle; however, desmin was also found in aggregates without αB-crystallin-associated immunoreactivity (Fig. 6*B*). To determine whether visualized αB-crystallin inclusions were indeed insoluble aggregates, we immunoblotted detergent-soluble and detergent-insoluble protein fractions from wild-type as well as αB-R120G heterozygous and homozygous tibialis anterior muscle. Consistent with the immunostaining results, insoluble αB-crystallin was only detected in αB-R120G heterozygous and homozygous mutant mouse muscle lysates. The insoluble αB-crystallin level was highest in the homozygous mutant mouse muscle (Fig. 6*C*).

## Discussion

Individuals with the αB-crystallin arginine 120 to glycine (R120G) mutation typically have one copy each of the mutant and wild-type allele. Over time, this mutation leads to the development of cataracts and DRM [12]. In this study, we generated knock-in mice with αB-crystallin R120G heterozygosity to elucidate the mechanism of cataract formation and DRM. The knock-in mutant mice developed myopathy and cataracts similar to human individuals carrying the αB-crystallin R120G mutation. The lens cataract and myopathic muscle in mutant mice and humans with the αB-crystallin R120G mutation shared common pathological features and molecular mechanisms. We observed significant α-crystallin aggregation in the lenses of mutant mice, which increased with cataract severity. We also found that the molecular weight of β- and γ-crystallin fractions from the mutant lenses was higher than the wild-type lenses (Table 3) because these peaks have more light scattering, implying that β- and γ-crystallin fractions aggregate more in heterozygous and homozygous mutant lenses. Our data also demonstrate an incremental increase in the lens γ-crystallin peak fraction with the αB-R120G mutation, suggesting that αB-crystallin may affect the expression of γ-crystallin. Our results appear to corroborate a previous report indicating that α-crystallin binds to specific regions of DNA in mouse γD/E crystallin genes [45]. We found no evidence to confirm that the increase in γ-crystallin is because of an increase in α- or β-crystallin fragments. Whether the increase is caused by increased expression or decreased degradation of γ-crystallin in the heterozygous and homozygous αB-R120G mutant lenses remains to be determined. A small increase in γ-crystallin by gel permeation chromatography has been reported in another mouse model for cataracts [44].

Additionally, the mutant mice accumulated αB-crystallin-vimentin aggregates in lens cells and αB-crystallin-desmin aggregates in muscle cells. The mechanism of lens opacification likely involves a change in the interaction between vimentin and αB-crystallin [31]. In heterozygous lenses, the interaction between vimentin and αB-crystallin was elevated, even in the absence of significant opacification. This increased interaction and aggregate formation were also observed in the lens epithelial zone of the mutant lenses by immunofluorescence analysis, consistent with published studies using cultured cells [28], [31], [46]. These results indicate that the interaction between the intermediate filament protein vimentin and αB-crystallin may be a precursor to the development of opacities in the mutant lenses. Notably, the mice used in this study were of a mixed 129Sv and C57BL6 background, and the129Sv strain of mice is known to lack a lens-specific intermediate filament known as the beaded filament [47], although mice lacking the beaded filament do not develop significant opacities and changes in vimentin levels. It would be interesting to determine whether cataract development in αB-R120G knock-in mice is altered in mouse strains that have the full complement of the beaded filaments.

αB-crystallin is expressed in the cornea [7] where it is important for corneal clarity. Consistent with this, a proportion of the R120G mutant mice developed corneal opacities. However, it is unclear why only a proportion of animals showed this effect. More of the animals are likely to develop corneal opacities as they age, and studies are in progress to assess this possibility. Patients with mutations in the αA-crystallin gene develop similar microcornea and corneal opacities [48], [49], substantiating our findings in knock-in mice and indicating that αB-crystallin also plays a critical role in the maintenance of corneal clarity. A small but significant fraction of the αB-R120G mutant mice had smaller eyes than wild-type littermates, although this small eye phenotype was not as prominent as in homozygous αA-R49C mutant mice [40], [50].

DRMs are a growing class of skeletal muscle disorders caused by mutations in desmin, αB-crystallin, Z-band alternatively spliced PDZ motif, myotilin, filamin C, and Bag3 [51]. Individuals with DRM typically develop late-onset progressive distal and proximal muscle weakness. Muscle biopsies from DRM patients have characteristic desmin inclusions. αB-R120G heterozygous and homozygous mutant mice recapitulate many of the pathologic features observed in DRM patients, including myopathy, desmin aggregates, and mitochondrial pathology [52]. These mice will be invaluable for expanding our understanding of how protein aggregates lead to skeletal muscle dysfunction.

As mentioned above, DRM is characterized by aggregates of intermediate filament (IF) proteins that are associated with αB-crystallin, such as desmin [12]. In cultured cells, heat shock and drug treatment induce collapse of IFs and associated Hsp27 and αB-crystallin, demonstrating that these small heat shock proteins are not sufficient to prevent filament collapse, and suggesting that the purpose of this association is more than just structural [31]. Inclusion of αB-crystallin prevents IF gel formation *in vitro* but not filament assembly, suggesting that one of the major functions of the association of small heat shock proteins with IFs is to help regulate the interactions that occur between filaments in their cellular networks [31]. Investigators have suggested that αB-crystallin does not alter the polymerization state of IF proteins in primary astrocytes, but ectopic expression of αB-crystallin in the absence of stress can modify the organizational state of IF and αB-crystallin can function as an IF debundling protein [53]. The sequence and structural domains that mediate αB-crystallin interactions with IFs include peptide residues 113–120, the region in the C-terminal domain of human αB-crystallin that includes the αB-R120G that causes human hereditary cataracts and myopathy, suggesting dysregulation of IFs may contribute to hereditary cataracts and DRM [54].

Wild-type αB-crystallin plays a beneficial role in the formation of desmin filament networks in muscle [31], [55]. In humans, and perhaps also in mice, the promotion of desmin filament aggregation by αB-R120G mutant protein may be repressed at a young age by the expression of competing wild-type αB-crystallin, which could explain the late onset of myopathies caused by αB-crystallin mutations [12], [28].

In contrast to the knock-in mice reported in this study, αB−/− knockout mice develop degeneration of certain skeletal muscles, most notably axial muscles and the tongue with relative sparing of the limb muscle, at about 65 weeks of age [8]. This onset occurs much later than the phenotypic changes observed in our knock-in mice, which were present as early as 26 weeks. This suggested that the DRM phenotype associated with the αB-R120G crystallin mutant is not caused merely by the loss of mutant protein function or a dominant-negative suppression of wild-type αB-crystallin function. Similarly, αB−/− knockout mice did not develop lens opacities or elevated stress-induced light scattering, in dramatic contrast to the lens opacities observed as early as 3 weeks in the αB-R120G knock-in mice described here. The results of the present study indicate that the mutant αB-R120G protein exhibits a gain of toxic function that explains the autosomal dominant nature of the αB-R120G-associated cataracts and myopathic disease.

Desmin-related cardiomyopathy has been recapitulated in transgenic mice by cardiac-specific expression of the αB-R120G protein [56]. These mice develop severe cardiomyopathy with early death at 28 weeks in a gene dosage-dependent manner. We did not observe a similar elevated mortality in αB-R120G crystallin knock-in mice. This may be because of expression of αB-R120G crystallin at endogenous levels, whereas previous studies [56] have overexpressed αB-R120G crystallin in the target tissue. Cardiac chaperone dysfunction perturbs cardiac mitochondrial architecture and impairs mitochondrial function [32]. These changes ultimately lead to cardiomyocyte death, dilation, and heart failure [32]. In another study, increased glucose-6-phosphate dehydrogenase expression was sufficient to cause cardiomyopathy in transgenic mice, which may be an additional mechanism underlying αB-R120G-associated cardiomyopathy [33]. Other *in vitro* studies have suggested that the intracellular aggregation of desmin and mutant αB-R120G in DRM may be caused by impaired αB-crystallin function and loss of its native supramolecular organization [55]. Investigators have also suggested that wild-type αB-crystallin might co-oligomerize with αB-R120G and prevent the formation of aggresomes. Other chaperones such as HSP70 or HSPB8 have also been shown to reduce the frequency of aggregate formation *in vitro* [55], [57]. Although the molecular basis of cataract and DRM development is not fully understood, the αB-R120G knock-in mice are a useful model for identifying the effects of molecular mechanisms that affect intermediate filament aggregation and lead to cataract formation and DRM.

In summary, we have generated a knock-in αB-R120G mouse model to study the mechanisms of human hereditary cataract development and have demonstrated that these animals begin to develop cataracts and muscle dysfunction at a young age. This multi-system model recapitulates many features of human hereditary cataracts and DRM. We have shown that the lens and muscle pathologies share many features, including increased insolubility of αB-crystallin with gene dosage, and increased co-aggregation of αB-crystallin with intermediate filament proteins. Thus, the αB-R120G knock-in mouse model is an essential tool for understanding the mechanisms underlying the development of hereditary cataracts and myopathy in individuals with αB-R120G mutations.

## Materials and Methods

### Generation of knock-in mice

Knock-in mice were generated by homologous recombination in 129SvJ male embryonic stem (ES) cells (SCC-10), modifying the αB-crystallin gene (*cryab*) such that exon 3 contained the R120G mutation in one allele, while the second copy of the gene was wild type. A mouse genomic DNA clone containing the αB-crystallin gene derived from a 129Sv strain was generously provided by Dr. Eric Wawrousek. The 2.3-kb 5′ arm was inserted into a cloning plasmid containing the neomycin cassette. The target nucleotide in exon 3 was mutated from A to G by site-directed mutagenesis (QuickChange kit; Stratagene, Santa Clara, CA, USA), the 2.8-kb 3′ arm of mouse *cryab* was cloned into the plasmid (**Fig. 1**), and then the plasmid was electroporated into ES cells. ES cell selection, colony picking, freezing, expansions, and cryopreservation of homologous recombinant clones expressing the R120G mutant αB-crystallin gene were performed at the Washington University ES Cell Core facility. Clones positive for neomycin were selected with G418, and 150 ES cell colonies were screened for correct gene targeting by Southern blot analysis. The A to G mutation was verified by sequencing the genomic DNA of positive ES clones (Fig. 1*B*). One clone (clone 24) was correctly targeted and used to generate knock-in mice (Fig. 1*C*). Correct insertion of the knock-in allele was tested by probing the 5′ and 3′ ends of *cryab* in the plasmid construct, and using primers outside *cryab*. ES cells positive for the mutation were karyotyped, electroporated with the Turbo-Cre plasmid to remove the floxed neomycin cassette, and karyotyped again. ES cells were then injected into C57BL6 blastocysts, which were then implanted into pseudopregnant ICR (imprinting control region) females. Chimeric founders were mated with wild-type C57BL6 mice. Genotyped progeny that were positive for germline transmission were bred. First generation offspring that inherited the targeted allele with neomycin were subsequently mated with C57BL/6J mice. The primers used to genotype the mice were 5′-GGA TTA GGA CGA ACA TGG CTT CAT CTC CG-3′ (forward) and 5′-CCA CCG ATG TCC TAT TTA CTG TCC TGC G-3′ (reverse).

Heterozygous offspring within each mating scheme were subsequently bred to yield homozygous mice. PCR genotypes of heterozygous and homozygous knock-in mice after deletion of the neomycin cassette are shown in Fig. 1*D*. Mice were maintained at Washington University by trained veterinary staff in the Division of Comparative Medicine. All protocols and animal procedures were approved by the Washington University Animal Studies Committee (protocol number 20090031).

### Slit lamp examination and recording

Slit lamp biomicroscopy was performed on non-anesthetized mice. Pupils were dilated with a mixture of 10% phenylephrine hydrochloride and 1% tropicamide (Alcon, Fort Worth, TX, USA). After 3 min, each mouse was positioned directly facing the slit lamp, holding the animal gently by the scruff of the neck. Next, both eyes of the animals were examined. Finally, the knock-in mice were examined at postnatal ages of 3–8 weeks, 12–20 weeks, and 40–56 weeks.

### Assessment of lens opacity and corneal changes

Cataract formation was scored by slit lamp biomicroscopy as follows: stage 0, clear lens; stage 1, loss of normal appearance of anterior, nuclear, and posterior lenses as well as prominence of y-suture line; and stage 2, discrete anterior changes accompanied by distinct nuclear opacity. The following changes were evident at 3–8 weeks: increased involvement of nuclear and cortical regions of the lens accompanied by increasing opacity (stage 3) and completely mature cataract involving the cortex and nucleus (stage 4). Stage 4 characteristics were observed in some of the homozygous lenses by 20 weeks. For each time point and strain, at least four animals were used, unless otherwise stated. The p-values were determined using unpaired Student's *t*-test.

### Analytical chromatography and analysis

High performance liquid chromatography (HPLC)-GPC was performed using a VE 1122 pump with a VE 7510 degasser (Viscotek/Malvern, Houston, TX, USA) that was equipped with a TDA302 triple detector system that measured the RI, multi-angle laser light scattering, and viscosity. The latter was supplemented with a model 2501 UV detector set at 280 nm. Two columns were connected in series: a Poly [Analytic] PAP-402.5 (Lausanne, Switzerland) and a G4000PWXL (Tosoh Biosep, Montgomeryville, PA, USA). Viscotek Omnisec software was used to calculate the RI area, weight-averaged molecular weight, intrinsic viscosity, and hydrodynamic radius. Three-month-old mouse lens proteins were analyzed on this column system. Samples were injected into a volume of 100 µl. The flow rate was 0.8 ml/min and the column buffer contained modified Dulbecco's phosphate-buffered saline (PBS) without CaCl_2_ and MgCl_2_ (Sigma-Aldrich, St. Louis, MO, USA). Samples from the column were then collected for further analysis. First, the amount of protein present in the wild-type, heterozygous mutant, and homozygous mutant lens samples was calculated using the RI area from the initial run. Samples of each condition were then re-run using approximately equal amounts of total protein. Column fractions of each condition were collected at 1-min intervals (800 µl/tube). To further analyze changes in α-crystallin fractions in older mouse lenses, we also performed GPC using A4000PWXL and G4000PWXL columns in series. All other conditions were the same as described above. Two to four lenses were analyzed per age and genotype. Chromatography runs were repeated three times.

### Molecular mass standards

Protein standards with known molecular masses were used to standardize and validate the column and detectors. Identical conditions were then used for separating lens crystallins. The primary standard was high purity bovine serum albumin (A0281, 67 kDa; Sigma-Aldrich). Other molecular weight protein standards obtained from Amersham Biosciences GE Healthcare (Piscataway, NJ, USA) were: dextran blue (2,000 kDa), thyroglobulin (669 kDa), ferritin (440 kDa), catalase (232 kDa), aldolase (158 kDa), ovalbumin (43 kDa), carbonic anhydrase (35 kDa), and ribonuclease A (13.7 kDa).

### Assessment of major intrinsic protein (MIP/AQP0) by immunofluorescence

Lenses were embedded in paraffin and 4-µm sections, stained with a polyclonal antibody to MIP (Alpha Diagnostics International) and an Alexa-568-conjugated secondary antibody, were visualized by confocal microscopy using a Zeiss 510 confocal microscope (Zeiss, Jena, Germany). Four lenses per genotype and two sections per lens were analyzed.

### Assessment of vimentin-αB-crystallin interactions by immunoprecipitation and immunoblotting

A co-immunoprecipitation assay was used to investigate the effect of the αB-R120G mutation on the association of vimentin with αB-crystallin. Mouse lenses were extracted in lysis buffer (50 mM Tris-HCl (pH 7.4), 150 mM NaCl, 1 mM EDTA, 0.1% sodium dodecyl sulfate (SDS), and protease inhibitor cocktail (Sigma-Aldrich), lysed for 5 min on ice, and then centrifuged for 10 min at 10,000× *g*. Supernatants were treated with a polyclonal primary antibody against vimentin (a gift from Dr. Paul Fitzgerald) conjugated to amino-link plus coupling resin (Pierce Biotechnology, Rockford, IL, USA) according to the manufacturer's protocol. Control rabbit serum conjugated to the resin was used as a control for immunoprecipitation. Immunoprecipitated proteins were washed by centrifugation at 4°C for 3 min, eluted with 200 µl of buffer containing 100 mM glycine (pH 2.5) for 10 min at room temperature, and then centrifuged at 10,000× g for 5 min. The resulting supernatants were neutralized with 1.25 µl potassium phosphate solution (pH 9.0). Proteins were resuspended in SDS-polyacrylamide gel electrophoresis (PAGE) sample buffer, and then analyzed by SDS-PAGE as described previously [58]. Immunoblot analysis with antibodies against αB-crystallin (Assay Designs, Ann Arbor, MI, USA) was performed. Two to four lenses were analyzed per genotype. Densitometric analysis was performed as described previously [59] and averages of two independent experiments were determined.

### Quantitative grip strength measurements

Strength testing consisted of five separate measurements using a trapeze bar attached to a force transducer that recorded the peak generated force (Stoelting, Wood Dale, IL, USA). Mice instinctively grabbed the bar with their forepaws and continued to hold while being pulled backwards by the tail, releasing only when unable to maintain grip. Five consecutive measurements were recorded: the highest and lowest measurements were discarded and the three remaining measurements were averaged to obtain the strength score. For each time point and strain, at least four animals were used, unless otherwise stated. The p-values were determined using paired Student's *t*-test.

### Muscle histology

Freshly isolated skeletal muscle was mounted using tragacanth gum, and then quick frozen in liquid nitrogen-cooled isopentane. Samples were then stored at −80°C until sectioning. Frozen biopsy samples were cut into 7-mm-thick sections. Skeletal muscle was taken from the quadricep, tibialis anterior (TA), and soleus/gastrocneumius muscles. Histochemistry and immunohistochemistry were performed as previously described [60]. Antibodies used were mouse anti-desmin (1∶200; Dako), mouse anti-FK2 (ubiquitin; 1∶500), and rabbit αB-crystallin (1∶100; Stressgen). Two to three mice were analyzed per genotype, and at least three sections per mouse were analyzed.

### Analysis of muscle αB-crystallin solubility

Fresh TA muscle was collected and flash frozen in liquid nitrogen. Tissue (20 mg) was homogenized in 250 µl of 2% SDS-radioimmunoprecipitation assay buffer (50 mM Tris-Cl (pH 8.0), 150 mM NaCl, 1% NP-40, 0.5% sodium deoxycholate, and 2% SDS) and protease inhibitor cocktail (Sigma-Aldrich). Homogenates were precleared with a 30-sec low speed spin and an aliquot of the supernatant was collected and named the total fraction. The additional supernatant was centrifuged at 100,000× *g* for 30 min at 4°C and this supernatant was collected and named the soluble fraction. The pellet was then sonicated on ice after the addition of 150 µl of 5 M guanidine-HCl and re-centrifuged at 100,000× *g* for 30 min at 4°C. This supernatant was removed and named the insoluble fraction. The insoluble fraction was precipitated by adding an equal volume of 20% trichloroacetic acid (Sigma-Aldrich) and incubated on ice for 20 min. Samples were then centrifuged at 10,000× *g* for 15 min at 4°C and the resulting pellet was washed twice with ice-cold acetone. Residual acetone was removed by drying tubes at 95°C and the samples were resuspended in 50 µl of 5% SDS in 0.1N NaOH. The protein concentrations of all samples were determined using a BCA protein assay kit (Pierce). Each sample (30 µg) was analyzed by Western blotting for each fraction as previously reported [60] using anti-αB-crystallin (1∶2500; Stressgen) and anti-desmin (1∶2500; Dako).
